# Deterministic Propagation Modeling for Intelligent Vehicle Communication in Smart Cities

**DOI:** 10.3390/s18072133

**Published:** 2018-07-03

**Authors:** Fausto Granda, Leyre Azpilicueta, Cesar Vargas-Rosales, Mikel Celaya-Echarri, Peio Lopez-Iturri, Erik Aguirre, Jose Javier Astrain, Pablo Medrano, Jesus Villandangos, Francisco Falcone

**Affiliations:** 1 Electrical and Electronic Engineering Department, Universidad de las Fuerzas Armadas ESPE, Sangolquí 171-5-231B, Ecuador; flgranda@espe.edu.ec; 2 School of Engineering and Sciences, Tecnologico de Monterrey, Monterrey 64849, Mexico; cvargas@itesm.mx (C.V.-R.); mikelcelaya@gmail.com (M.C.-E.); 3 Electrical and Electronic Engineering Department, Public University of Navarre, 31006 Pamplona, Spain; peio.lopez@unavarra.es (P.L.-I.); erik.aguirre@unavarra.es (E.A.); francisco.falcone@unavarra.es (F.F.); 4 Mathematical Engineering and Computer Science Department, Public University of Navarre, 31006 Pamplona, Spain; josej.astrain@unavarra.es (J.J.A.); pablo.medrano@unavarra.es (P.M.); jesusv@unavarra.es (J.V.)

**Keywords:** Smart Cities, Wireless Sensor Networks (WSN), 3D Ray-Launching, Vehicular Ad-Hoc Networks (VANET), Vehicle-to-Infrastructure communication (V2I)

## Abstract

Vehicular Ad Hoc Networks (VANETs) are envisaged to be a critical building block of Smart Cities and Intelligent Transportation System (ITS) where applications for pollution, congestion reduction, vehicle mobility improvement, accident prevention and safer roads are some of the VANETs expected benefits towards Intelligent Vehicle Communications. Although there is a significant research effort in Vehicle-to-Infrastructure (V2I) and Vehicle-to-Vehicle (V2V) communication radio channel characterization, the use of a deterministic approach as a complement of theoretical and empirical models is required to understand more accurately the propagation phenomena in urban environments. In this work, a deterministic computational tool based on an in-house 3D Ray-Launching algorithm is used to represent and analyze large-scale and small-scale urban radio propagation phenomena, including vehicle movement effects on each of the multipath components. In addition, network parameters such as throughput, packet loss and jitter, have been obtained by means of a set of experimental measurements for different V2I and V2V links. Results show the impact of factors such as distance, frequency, location of antenna transmitters (TX), obstacles and vehicle speed. These results are useful for radio-planning Wireless Sensor Networks (WSNs) designers and deployment of urban Road Side Units (RSUs).

## 1. Introduction

Vehicle-to-Infrastructure (V2I) is the next generation of Intelligent Transportation Systems (ITS). In this sense, Smart Cities must be able to take advantage of its applications and benefits on transportation operations according to main areas of research goals identified by the U.S. Department of Transportation (U.S. DOT) (https://www.its.dot.gov): safety, mobility, efficiency, productivity, energy and environmental impacts, and customer satisfaction, offering to travelers more mobility options and access opportunities. To meet these goals, ITS technologies would be integrated into other operational areas of the Smart Cities as is proposed in the Smart Columbus program [1], where the advancements in connected vehicles (CV), automated vehicles (AV), and electric vehicles are integrated with the data from various sectors and sources to simultaneously power these technologies while leveraging the new information they provide. These ITS technologies can be seen as a part of a holistic or wider mobility approach where some sustainable transportation strategies such as strengthening urban road construction, improving operational efficiency and private traffic restriction have been proposed as sustainable strategies for transportation development [2]. 

Countries such as Canada, the United States of America (USA), Mexico, European nations, the Middle East and Japan are pioneers and adopters of ITS research and applications into vehicles, infrastructure and traffic management where governmental agencies are strongly involved and play a key role in delivering safe and efficient transportation. As an overview, in Canada, Transport Canada (http://www.tg.gc.ca) is focused on the development of relevant transportation policies and legislation, while in USA, the U.S DOT aims to ensure a fast, safe, efficient, accessible and convenient transportation system that meets U.S. vital national interests and enhances the quality of life of the public. In Mexico, the Mexican Institute of Transport (http://www.imt.mx) conducts research projects around public and private transport and implements programs aimed at developing adequate human capital for the transportation sector. 

V2I communication is the bi-directional wireless exchange of data (control and information) between vehicles and Road Side Units (RSUs), wirelessly providing information such as advice from the infrastructure to the vehicle that inform the driver of safety, mobility, or environment-related conditions [3]. In 1999, the U.S. Federal Communication Commission (FCC) allocated a 75 MHz of licensed spectrum at 5.9 Ghz to be used as V2V and V2I communications known as Dedicated Short Range Communications (DSRC), and the IEEE 802.11p standard [4], part of the Wireless Access in Vehicular Environments (WAVE) initiative [5], was developed to operate at this 5.9 Ghz band, with 75 Mhz bandwidth and seven 10 Mhz channels. This dedicated bandwidth provides a low-latency, short-to-medium-range wireless communications medium that permits fast and reliable data transmissions at high transmission rates, critical for safety applications. 

In this context, the U.S. DOT announced new V2I guidance [6] that will boost the ongoing private [7], academic [8] and governmental [9] research efforts and projects in the area of ITS including applications ranging from safety critical [10,11,12] and traffic management [13,14] up to gaming and in-car multimedia streaming [15]. Some of V2I applications reported in the Transit Vehicle-to-Infrastructure (V2I) Assessment Study [16] are: Transit Vehicle-Pedestrian/ Cyclist Crossing Warning, Transit Stop/Station Pedestrian Safety, Transit Vehicle and Center Data Exchange Application, Transit Traveler Information Infrastructure Application, Red Light Violation Warning (RLVW), Left Turn Assist (LTA), Stop Sign Violation Warning (SSVW), Reduced Speed Zone Warning (RSZW), Pedestrian in Signalized Crosswalk Warning (PCW) etc. A proposed application aimed at assisting transit vehicle maneuvers and detours to handle special events at strategic locations to perform dynamic information collection/dissemination, is the V2I Portable Infrastructure which features the concept of portable infrastructure such as portable RSUs and signage. 

The design of accurate propagation models for realistic V2I enabling must take into account some unique characteristics in terms of antenna heights, placement of the RSUs, environment type (e.g., urban, suburban, highway) and specific considerations for small-scale fading due to particular location of antennas [17]. Urban environments are characterized by the combination of different object types such as buildings, vehicles, pedestrians, vegetation etc., as well as their number, size, and density, that have a profound impact on radio propagation [18]. Some propagation impairments such as reflection from, diffraction around and transmission loss through objects (influence of vegetation, building entry loss, cars, trees, pedestrians, etc.), and external environment, give rise to issues such as Quasi-Line-of-Sight (QLoS), Non-Line-of-Sight (NloS), temporal and spatial variation of path loss and multipath effects from reflected and diffracted components of the wave. The rapid changes in radio propagation conditions are one of the main challenges for channel modelling in vehicular communications. 

Channel modeling approaches can be roughly classified into four categories [19]: (1) empirical (they require calibration but offer rapid results); (2) stochastic (characterize the channel from a frequency selection perspective); (3) geometry-based stochastic (commonly used for propagation prediction in mobile communication); and (4) deterministic (used in propagation prediction of specific environments). While stochastic and geometry-based stochastic could fail to characterize significant surrounding obstacles (foliage, lamppost, pedestrians, etc.) and V2I empirical tests provide useful insight for specific in situ scenarios [20], the deterministic ray launching simulators are suited for vehicular propagation analysis of large-size scenarios yielding a reasonable tradeoff between accuracy and computational cost. The principle of ray launching approaches is that a consistent number of path rays follows from the transmitter to the receiver over the direct, reflected and diffracted rays. Some current state-of-the art simulators focus mainly on V2V or V2I communications operating in a cellular sense and, although the literature includes many propagation models and channel simulators for V2V systems [21,22], there is a need for further studies to investigate V2I propagation using 3D deterministic tools in complex environments such as urban ones. 

In this work, an in-house 3D Ray-Launching (3D-RL) algorithm (hybrid deterministic method) based on Geometrical Optics (GO), Geometrical Theory of Diffraction (GTD) and its extension the Uniform Theory of Diffraction (UTD) has been used to analyze the large-scale and small-scale signal propagation phenomena, which includes the analysis of Doppler Shift and Doppler Spread as time-changing statistics of the V2I channel in a three-dimensional (3D) urban scenario. Homogeneous clusters of information (in terms of data dispersions) were used to identify subtle variations in the large-scale and small-scale statistics considering factors as relative distance and position from the antenna transmitters (TX), geometry, density and electric properties of the obstacles and permit estimation of the variations in time and frequency of small-scale fading given the non-stationary nature of vehicular communications channel [23]. The statistical analysis concludes that Lognormal, Gamma and Weibull distribution can explain the fading behavior of this multipath environment where the amplitude of fading is highly affected by the data dispersion.

The remaining parts of the paper are outlined as follows: Section 2 presents the materials and methods used in this work, with a brief explanation of the 3D-RL technique, the description of the urban scenario considered for radio wave propagation characterization and the experimental measurements performed in the same scenario for the analysis of network parameters of the V2V and V2I communication links. Section 3 reports the large-scale and small-scale characterization of the urban vehicular scenario. Statistical analysis of radio wave propagation characterization is presented in Section 4. Section 5 shows a network parameter communication link analysis based on the experimental campaign of measurements performed in the same vehicular scenario, obtaining in this way a complete characterization (physical and network layer) of vehicular communication links. An application example is presented in Section 6, and conclusions and future work are summarized in Section 7.

## 2. Materials and Methods

### 2.1. The Ray Launching Technique

As stated before, an in-house 3D-RL algorithm has been used for the characterization of radio wave propagation in the considered urban scenario. The detailed operating mode of the algorithm has been previously published in [24]. It has also been validated in large indoor environments [25], intra-vehicle [26] and transportation systems [19,27,28]. The principle of the algorithm is that the complete 3D scenario is divided into a fixed number of different cuboids sizes. The position of the transmitter antenna is an input parameter of the algorithm; thus, rays are launched from the transmitter position with a fixed angular and spatial resolution, and propagation parameters are calculated for each cuboid along the ray path until the ray reaches the maximum number of reflections considered. The advantage of deterministic approaches is that it permits the consideration of all the obstacles within the environment, taking into account all the material properties, considering the conductivity and dielectric constant at the frequency range of operation of the system under analysis. Electromagnetic phenomena such as reflection, refraction and diffraction have been considered in the algorithm, leading to an efficient and robust technique already validated for the urban environment [27] and vehicular communications [19]. 

Different radiation patterns for the transceivers can be considered, as well as antenna parameters. The frequency of operation, number of multipath reflections, angular and spatial resolution, and cuboid dimensions are introduced. In addition, the frequency dispersive effects due to the movement of the vehicles can be analyzed by means of the Doppler shift and Doppler spread parameters, for different velocities of the vehicles and for the frequency under analysis. 

### 2.2. Urban Scenario Description

This subsection describes the selected urban scenario (google map (http://www.google.com/maps) with its 2D and 3D representation maps, reference points and main 3D-RL simulation parameters. 

**Figure 1:**
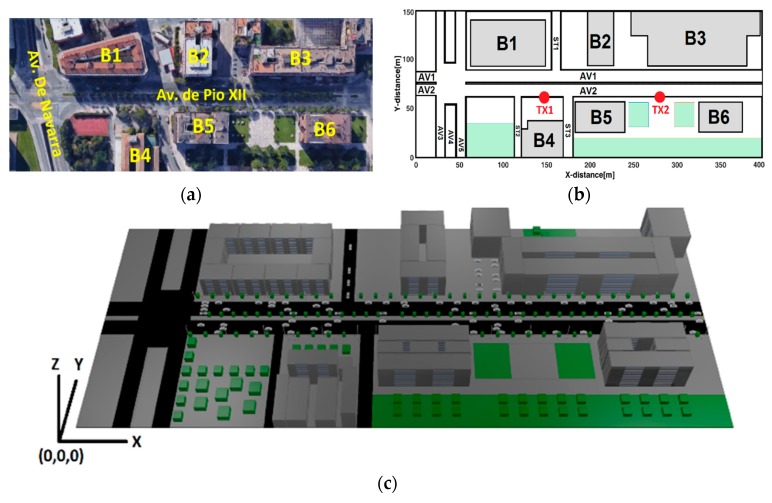
Urban scenario: (**a**) google map aerial view; (**b**) 2D-map schematic view; and (**c**) 3D-map frontal view.

Figure 1a shows the urban scenario aerial view, (b) is the 2D schematic (x, y) map and, (c) is the 3D frontal-view map used in the simulation. Table 1 identify the Cartesian coordinates (x, y, z) of interest points according its relative position from the origin (0, 0, 0). 

The modeled and simulated urban scenario is a replica of the Pío XII Avenue at the city of Pamplona, Spain (42°48′22.15 N, 1°39′39.14 W) and it was referenced using the Google Maps tool. The scenario encompasses an area of 1,320,000 m^3^ (400 × 150 × 22) and includes the typical elements of an urban environment (buildings, trees, cars, streetlights, pedestrians, etc.). Each side of the avenue has three lanes, and vehicles park parallel to the pavement on both sides. The parked vehicles coexist with metal and fiberglass garbage containers. The frequency for simulation was set up at 5.9 Ghz. Simulation parameters for the radio frequency (RF) characterization by means of the RL algorithm are summarized in Table 2.

The RST was chosen considering the receiver (RX) minimum sensitivity information provided for some manufactures of V2X (IEEE 802.11p) radio-communications products [29,30,31,32].

### 2.3. Experimental Measurements

**Figure 2:**
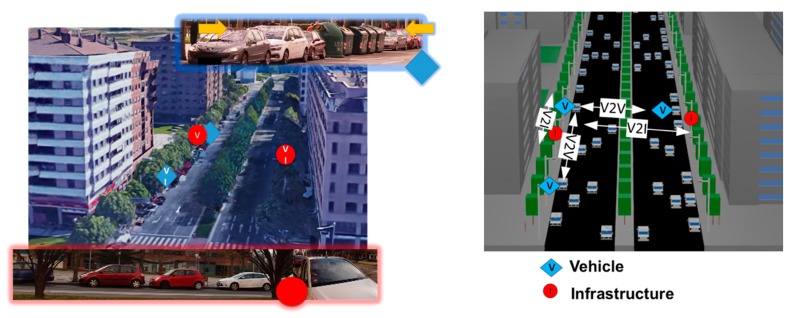
Location of vehicles, infrastructures, V2V and V2I communications links: real scenario (**left**) and schematic simulation scenario (**right**).

To complement the radio wave characterization assessment with network parameter analysis for V2V and V2I communication links, a set of experimental measurements have been developed in the same urban scenario. V2V and V2I communication links for four different configurations have been analyzed: V2V with the vehicles on the same and opposite sides, and V2I with the vehicle and infrastructure in the same and opposite sides. Figure 2 shows with a blue diamond the vehicles considered for the experiments, and with a red circle the location of the infrastructure (streetlight) located faced between them. The different communications links analyzed in the experiments are shown in Figure 2 (right).

**Figure 3:**
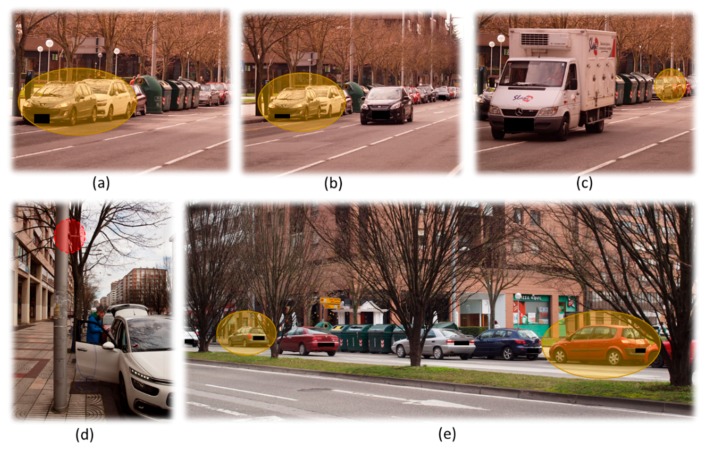
Working scenario: (**a**–**c**) location of the vehicles used for the experimentation remarked in yellow; (**d**) antenna fixed in the infrastructure remarked in red; (**e**) working scenario.

Figure 3 presents the vehicles involved in the experimentation re-marked in yellow (a, b, c and e), while the antenna fixed on the infrastructure is re-marked in red (d). Figure 3a–c show the traffic on the north east side of the avenue and the location of the three vehicles used for the experimentation. Figure 3d shows the antenna fixed to the streetlight which is connected to an Ubiquiti XtremeRange5 card placed into the vehicle and powered by its battery. Figure 3e shows that vehicles, trees, garbage containers and pedestrians coexist in the working scenario.

**Figure 4:**
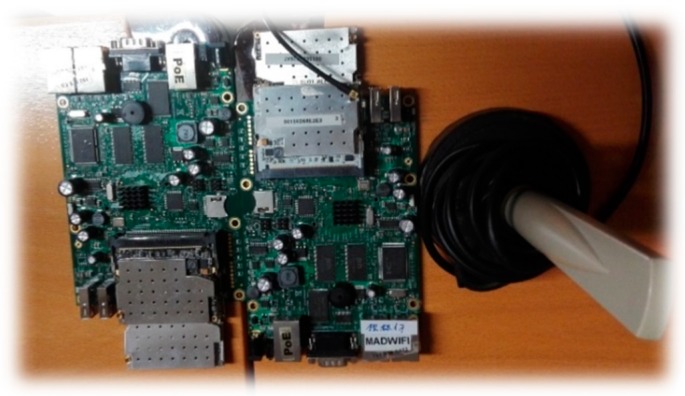
Two RB433 UAH MikroTik device, with two Ubiquiti XtremeRange2 and an Ubiquiti XtremeRange5 cards and the corresponding antenna.

The hardware used in the experiments has been two RB433 UAH MikroTik (MikroTik, Riga, Latvia) devices where two Ubiquiti XtremeRange cards (for 2.4 and 5 Ghz communication, respectively) are connected to each device (see Figure 4). The RB433 device is a 680 MHz CPU (AR7161) miniPC, with 128 MB of RAM, 3 miniPCI slots, a serial port and Openwrt as operating system. Power supply is provided to the RB433 device by a 12Volt DC vehicle car cigarette lighter power adapter and an AC/DC inverter. Although each RB433 device can use both 2.4 and 5.9 Ghz communication frequencies, due to the Ubiquiti XtremeRange2 (2.4 Ghz) and XtremeRange5 (5.9 Ghz) cards, in this work we have just considered the 5.9 Ghz band of the IEEE 802.11a standard. The average power consumption of the Ubiquiti XtremeRange5 card on transmission is 28 dBm. Antennas are magnetized allowing then to be fixed easily to both a vehicle roof and streetlights. Since the vehicles used for the experiment have energy-saving mechanisms, it has been necessary to keep the vehicle engines running during the experiment to ensure the uninterrupted power supply to the RB433.

Communications are analyzed by means of *iperf* (Iperf2, http://sourceforge.net/projects/iperf2), which is a widely used tool that allows measurement of network performance metrics such as throughput, packet loss and jitter. *Iperf* (v2), which has both client and server functionality, allows creation of data streams to measure the throughput between the two ends in one or both directions. During the experimentation, three MikroTik RB433 UAH devices have been used: one of the RB433 UAH devices acted as an *iperf* server, while the other ones acted as clients. The *iperf* server accepts multiple clients simultaneously. In our case, we have used three concurrent User Datagram Protocol (UDP) threads during the experimentation, and we have measured communication parameters such as bandwidth, transferred bytes, packet error rates (PER) and jitter. The choice of UDP is made for providing quick and easy communication between vehicles; it is more preferable to resend the same message several times than having to manage the confirmation of receipt by Transmission Control Protocol (TCP).

**Figure 5:**
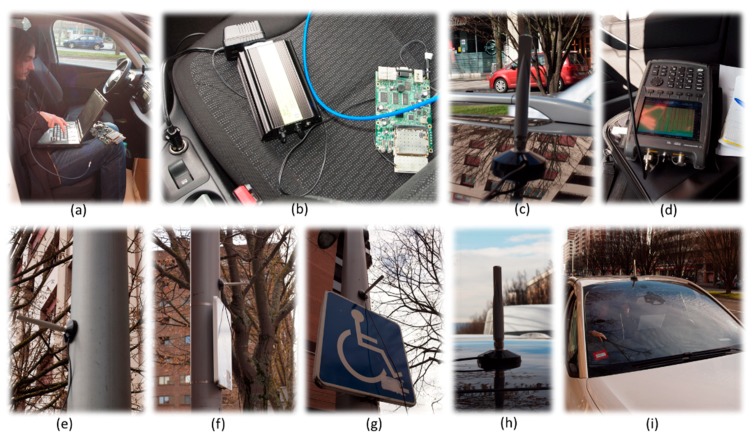
Communication devices for V2I and V2V communication: (**a**) laptop used to store the *iperf* traces; (**b**) AC/DC inverter plugged into the vehicle; (**c**) antenna placed on the vehicle; (**d**) spectrum analyzer; (**e**–**g**) antenna placed on the infrastructure; (**h**–**i**) antenna placed on the vehicle.

Figure 5 depicts the devices used to perform the tests, where (a) shows the laptop used to store the *iperf* traces, (b) shows the AC/DC inverter plugged into the vehicle, (c) (h) and (i) show the antennas placed on the roof of the vehicles, (d) is the portable spectrum analyzer used to analyze the communication V2V and V2I and (e) (f) and (g) show the location of the antennas used at the infrastructure (streetlight).

**Figure 6:**
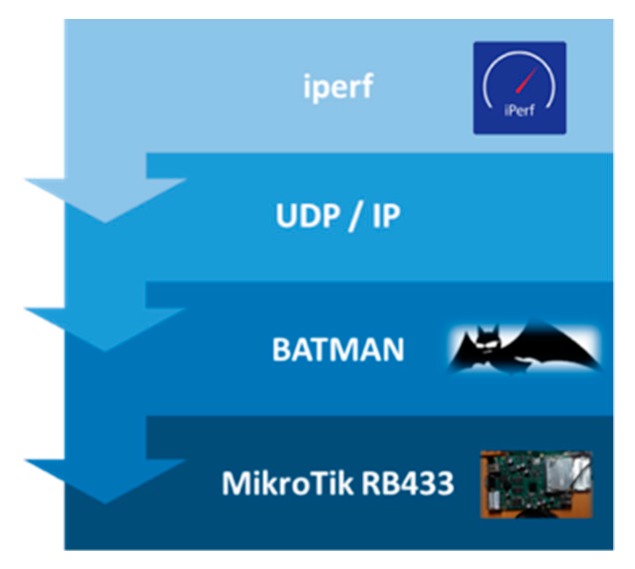
Communication protocol stack implemented for the experiments.

To provide and ensure VANET connectivity, we have selected the proactive distributed protocol BATMAN-ADV [33], which is the acronym of Better Approach To Mobile Adhoc Networking Advanced. It is a routing protocol for multi-hop mobile Adhoc networks that does not manage a centralized knowledge of the wireless network and does not need to consolidate information concerning network changes in every node in the network, since it does not try to determine the entire route at sending time. Each packet passing through each node of the network gets the next best jump at that time for its destination. Data is passed to the next neighbor at a given mac-address until it reaches its destination. We have placed the BATMAN-ADV protocol on each RB433 UAH device, and using *iperf*, we have analyzed communication flows between two static vehicles (V2V) and between a static vehicle and the infrastructure (V2I), as shown in Figure 3. Figure 6 shows the communication stack implemented for the experimental measurements. Network parameters obtained by the experimental measurements are presented in Section 5.

## 3. Simulation Results

### 3.1. Large Scale Spatial Path Loss

The Ray Launching technique is a well-recognized tool for the accurate estimation of the propagation model parameters useful in the radio-planning phase of the V2I communication projects. This large-scale spatial path loss characterization subsection presents a detailed spatial representation of the Received Signal Strength (RSS) as a function of the Euclidean distance from the transmitter (TX) to the receiver (RX) (TX height: 3.5 m, RX height: 1.5 m), along the avenues and streets. For illustrative purposes, the left (x-axis) and top (y-axis) distances from TX were prefixed with a negative sign, while the right and bottom distances remain positive. The RSS data generated by 3D-RL simulation is represented with a dotted line, while a Least Square (LS) fitting is represented with a dashed line. The LS fitting (first order polynomial) is robust for minimizing the effect of the outliers in the 3D-RL raw data and could be used for comparison purposes with theoretical path loss models. The Line-of-Sight (LoS) and Non-Line-of-Sight (NLoS) regions had been properly identified.

**Figure 7:**
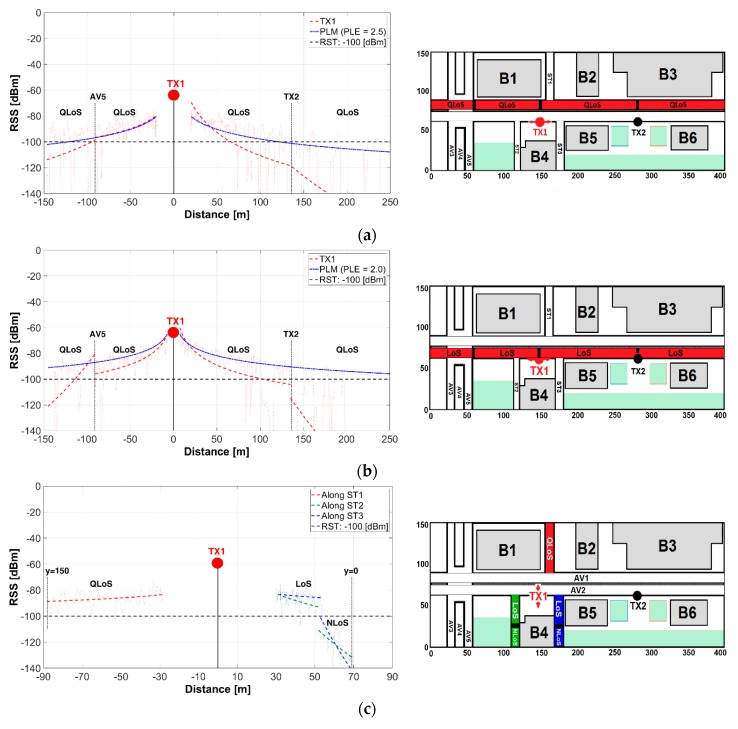
Spatial TX1-RSS Path Loss along: (**a**) AV1; (**b**) AV2; (**c**) ST1, ST2, ST3.

The TX1-RSS along AV1, AV2, ST1, ST2 and ST3 is depicted in Figure 7 while Table 3 summarizes the PLE and STD values along AV1, AV2, ST1, ST2 and ST3. Figure 7a illustrates the TX1-RSS along AV1 and its comparison with a Path Loss Model (PLM) [34]. This PLM describes the random shadowing effects over many measurements that have the same TX-RX separation, but different levels of clutter on the propagation path. The PLM received power $P_{r}$, is defined by Equation (1):

(1) $$P_{r}\left(d\right)\left[\mathrm{dBm}\right]=P_{t}\left[\mathrm{dBm}\right]+G_{t}\left[\mathrm{dB}\right]+G_{r}\left[\mathrm{dB}\right]-\left[\;\mathrm{PL}\left(d_{o}\right)\left[\mathrm{dB}\right]+10n\log10\left(\frac{d}{d_{o}}\right)\left[\mathrm{dB}\right]+X_{\sigma }\left[\mathrm{dB}\right]\right]$$

where:

- $P_{t}\;\mathrm{is}\;\mathrm{the}\;\mathrm{power}\;\mathrm{transmitted}\;\mathrm{by}\;\mathrm{the}\;\mathrm{TX}.$
- $G_{t}$ and $G_{r}$ are the gain of RT and RX respectively.
- $\mathrm{PL}\left(d_{o}\right)$ is the free space path loss (reference distance $d_{o}$ = 1 m), defined by Equation (2):
  (2) $$\mathrm{PL}\left(d_{o}\right)\left[\mathrm{dB}\right]=-10\log_{10}\left(\frac{\lambda^{2}}{{\left(4\pi \right)}^{2}d_{o}{}^{2}}\right)$$

where

- λ=c/f is the wavelength, where $c=3\;\times \;10^{8}\;m/s$ and $f=5\;\times \;10^{9\;}\mathrm{Ghz}$
- $d_{o}$ is the close-in distance from the TX (considered 1 m).
- $X_{\sigma }$ is a zero-mean Gaussian distributed random variable with standard deviation σ.
- d is the TX–RX distance.
- n, is the path loss exponent (PLE) and together with the standard deviation (STD) were estimated from the 3D-RL raw data, using Maximum Likelihood (ML) [35], according Equations (3) and (4):
  (3) $$\mathrm{PLE}=\frac{-\sum_{i=1}^{n}\left(P_{i}\left[\mathrm{dBm}\right]-P_{o}\left[\mathrm{dBm}\right]\right)\log_{10}\left(\frac{d_{i}}{d_{o}}\right)}{\sum_{i=1}^{n}10{\left(\log_{10}\left(\frac{d_{i}}{d_{o}}\right)\right)}^{2}}$$
  (4) $$\mathrm{STD}\;\left[\mathrm{dB}\right]=\frac{1}{\sqrt{n}}{\left\{\sum_{i=1}^{n}{\left[P_{i}\left[\mathrm{dBm}\right]-P_{0}\left[\mathrm{dBm}\right]+10\log_{10}\left(\frac{d_{i}}{d_{o}}\right)\right]}^{2}\right\}}^{\frac{1}{2}}$$

Factors such as distance, special type of street intersection (roundabout) and obstacles (trees and buildings) have significant impact on the propagated signal. The presence of a roundabout causes RSS degradation with RSS values below the RST, while a line of trees (height: 3 m) along AV1-AV2 causes Quasi-Line-of-Sight (QLoS) deriving the in signal fluctuation, which becomes evident in the reported STD values (Table 3, item a). The PLM is unable to predict the signal losses caused for the roundabout and line of trees, whose impact is greater to the extent that the TX-RX distance is increased.

Figure 7b depicts the RSS along AV2 where the LoS condition and shorter distance to TX1 permit a higher RSS and lower signal dispersion values when compared with AV1-RSS. The PLE and STD values, registered in Table 3, item b, are lower than that registered in item a, due to the aforementioned aspects. The expected TX1 coverage along AV2 is already 90m and 80m for its left and right side, respectively.

An additional TX1, TX2 coverage analysis is presented in the next subsection to clarify this estimation. Figure 7c depicts the RSS along ST1, ST2 and ST3 where the relative TX-RX closeness yields RSS values above the RST at ST1 (QLoS), the upper-area of ST2 (LoS) and all ST3 (LoS). The NLoS condition caused by B4 at the lower-area of ST2 and ST3, generate RSS values below the RST. Additional simulation, when TX1 or TX2 or TX1 and TX2 are transmitting, showed areas as the roundabout, AV3, AV4, AV5, ST2 (NLoS) and ST3 (NLoS) where the V2I communications are unfeasible.

### 3.2. Received Signal Strength

**Figure 8:**
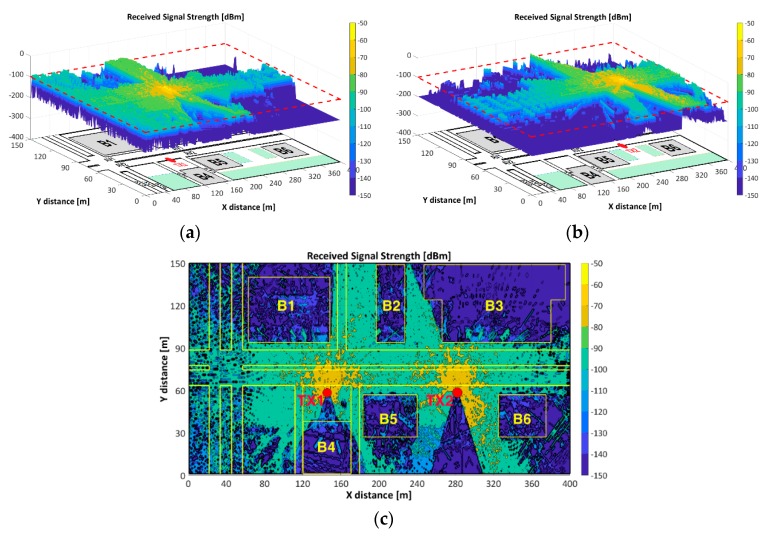
Spatial RSS and coverage map: (**a**) TX1 RSS; (**b**) TX2 RSS; (**c**) TX1&TX2 RSS

Figure 8 illustrates the RSS and coverage map of TX1 and TX2 at z-plane of 1.5m, the estimated RX antenna height of the passenger cars. Figure 8a,b show an RSS surf-plot of TX1 and TX2 respectively. Areas such as the roundabout, AV3, AV4, AV5 and the lower-area of ST2 and ST3 have RSS values below the RST. The dispersive effect caused by the trees, is not clearly defined in this view. Figure 8c depicts the jointly TX1 and TX2 contour-plot where RSS values above the RST are observed along AV1, AV2, ST1 and upper sector of ST2 and ST3 making feasible the V2I communication. The blocking of the signal due to the buildings is notorious (e.g., the building B4 causes the NLOS condition at ST2 and ST3).

#### Coverage

**Figure 9:**
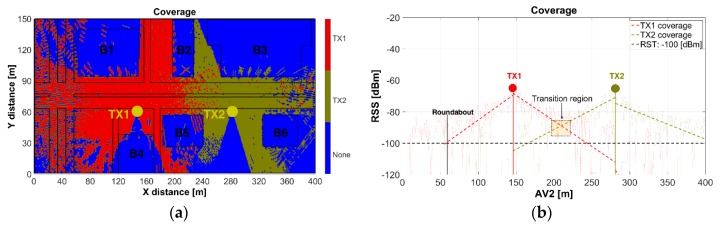
Coverage map: (**a**) scenario; (**b**) along AV2.

Figure 9 depicts the coverage map for the joint configuration of TX1 and TX2. The coverage map was based on the selection of the highest TX1 or TX2 RSS at each point of the scenario when both antennas are transmitting. RSS values below the RST were identified with “None”.

Figure 9a shows that the TX1 coverage encompasses AV1–AV2 (left-segment), ST1, ST2 (LoS) and ST3 (LoS), while the TX2 coverage embrace AV1–AV2 (right-segment). Figure 9b illustrates the AV2-RSS as function of the coverage-distance (x-axis) for each TX and, the transition region where the RX will define the point-to-point link either with TX1 or TX2, based on the sensing of the highest power received. Each TX has an approximate total coverage distance of 170m (asymmetric). The transition point is located approximately at the half distance between the TXs. The roundabout, AV3, AV4, AV5, ST2–ST3 (NLoS) is out of coverage.

The effectiveness of cooperative systems using RSUs for V2I communications is dependent on how efficiently RSUs are being deployed according Gonzalvez et al. in [20]. In this way, based on the simulated results, different TX placement configurations could be suggested to achieve RSS coverage levels above the RST; one of these configurations could be as follows:

- TX1 and TX2 in the same simulated placement to give coverage to AV1, AV2, ST1.
- Tree additional TXs: one for coverage of ST2, one for coverage of ST3 and, one for coverage the roundabout, AV3, AV4 and AV5 areas.

### 3.3. Multipath Metrics

This subsection presents the analysis of multipath metrics such as Power Delay Profile (PDP), Mean Excess Delay, Root Mean Square Delay Spread (RMS delay spread) and Coherence Bandwidth (CB). These are required parameters to develop a proper scheme for communications and determined for different environments to design some general guidelines for WSN deployment.

#### 3.3.1. Power Delay Profile (PDP)

**Figure 10:**
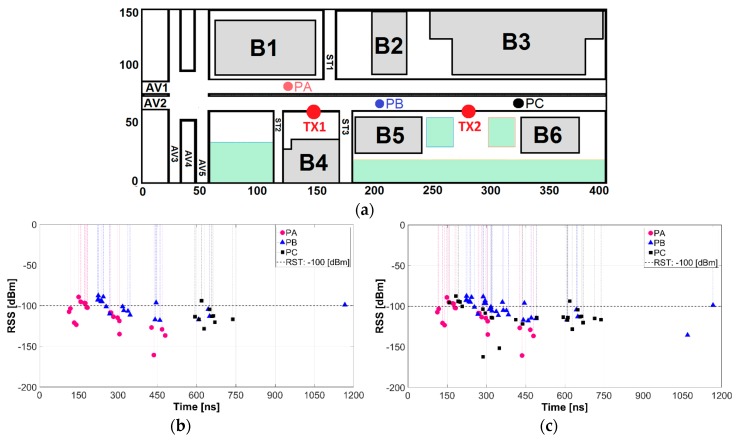
Power Delay Profile: (**a**) measured points; (**b**) transmitting TX1; (**c**) transmitting TX1 and TX2.

The PDP is used to calculate various multipath statistics (mean excess delay, RMS delay spread, maximum excess delay, CB) and quantifies the number and severity of power rays (echoes) in the wireless channel. Figure 5 illustrates the PDP at z-plane of 1.5 m for 3 different locations represented in Figure 10a, while Figure 10b,c depicts the PDP when TX1 or both TX1 and TX2 are transmitting. Figure 10b shows many power rays (echoes) in a time span of 0 to 1200 ns showing a high dispersive nature of this environment causing reflected, refracted, and diffracted rays that arrive to PA, PB and PC points. The highest density of rays arrives to PA which is in LoS and closest (first ray arrives at 100 ns) to TX1; conversely the lowest density is at PC, which farthest from TX1. Figure 10c illustrates the increase in the number of power rays arriving to PB and PC as a contribution effect of TX2 to the jointly PDP mainly at the transition region (refer to Figure 9b). The presence of power rays with low RSS values are mainly those reflected and diffracted by obstacles and the line of trees among AV1 and AV2.

#### 3.3.2. Mean Excess Delay, RMS Delay Spread, and Coherence Bandwidth (CB)

The mean excess delay (the first moment of the PDP) and the RMS delay spread (the square root of the second central moment of PDP) quantify the time dispersive properties of multipath channels while the Coherence Bandwidth (CB) is the range of frequencies over which two frequency components have a strong potential for amplitude correlation [34]. The NLoS scenarios have much higher mean excess delay than LoS scenarios given that the transmitted signals will encounter many reflections along their path to the receiver.

**Figure 11:**
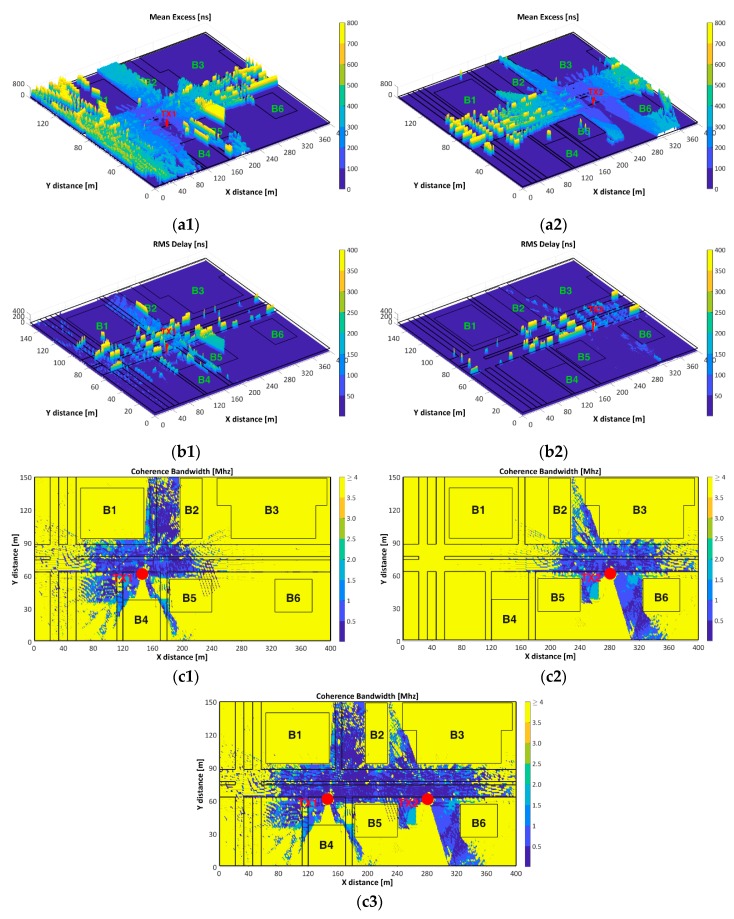
(**a1**–**a2**) Mean Excess Delay; (**b1**–**b2**) RMS delay spread; (**c1**–**c3**) Coherence Bandwidth.

Figure 11 shows the mean excess delay, RMS delay spread and CB (for frequency correlation of 0.9). Figure 11(a1,a2) depict the mean excess delay for TX1 and TX2 respectively, where higher values in the extent of distance is increased from the TX are observed, while lower values are related to low RSS due to the blocking of the propagated signal by concrete walls (signal absorption) and glass windows (signal reflection) of the buildings. The roundabout, AV3, AV4, AV5, ST3 and the rightmost segment of AV1–AV2 display the highest mean excess delay for TX1, while the leftmost segment of AV1–AV2 display this behavior for TX2.

Figure 11(b1,b2) illustrate the RMS delay spread. Lowest values keep a close relationship with lowest values of RSS due to the NLoS conditions. Some factors such as the presence of obstacles (trees, lampposts, cars) and the LoS conditions cause high RMS delay spread values near the TXs. As the TX-RX distance is increased, the amplitudes of the reflected signals relative to the direct path become larger, and this produces the increase of the mean excess and RMS delay spread as is depicted in Figure 11a,b.

Figure 11(c1–c3) depict the CB values of TX1, TX2 and TX1 and TX2 respectively. High CB values, in areas such as AV3, AV4, AV5, ST2–ST3 (NLoS), etc., correspond with low RMS delay spread (given its inverse relationship) and high CB could be considered as an indicator of channel availability for TXs other than TX1 or TX2. On the other hand, low CB values are present at the vicinity of the TXs due to its multipath environment, which means high channel occupancy. Figure 11(c3) illustrates a superimposed CB contour map of TX1 and TX2 to gain insight of the joint CB and low CB values along AV1–AV2 and ST1 are observed. The CB analysis must be complementary with the analysis of RSS, and coverage and RMS delay spread mentioned above.

#### 3.3.3. Doppler Spread (B_D_) and Doppler Shift (fd)

Doppler Spread (B_D_) and coherence time (T_C_) describe the time-varying nature of the channel, caused by either relative motion between the mobile and base station or by the movement of objects in the channel, in the small-scale region. B_D_ is defined as the range of frequencies (spectral broadening) over which the received Doppler Spectrum is essentially non-zero (if the baseband signal bandwidth is much greater than B_D_, the effects of Doppler spread are negligible at the receiver—slow fading channel). Doppler Shift (fd) is the Doppler spectrum of the received signal in the range fc – fd to fc + fd, where fc is the transmitted frequency [34].

**Figure 12:**
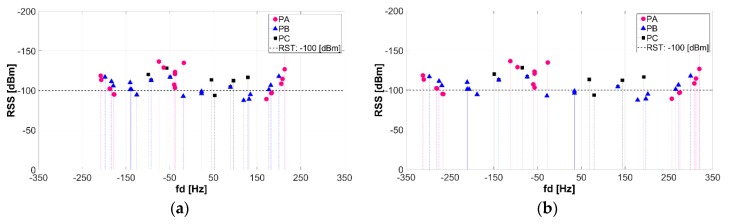
TX1-Doppler Shift (fd): (**a**) car velocity of 40 km/h, (**b**) car velocity of 60 km/h.

Figure 12 shows the effect of the relative velocity on the maximum Doppler Shift, which is when Cosθ = 1 (θ is the angle between the direction of motion of the mobile and direction of arrival of scattered waves). Figure 12a,b depicts Doppler Shift with respect to TX1 of three different cars PA, PB and PC (see Figure 10a) which are traveling at constant velocities (v) of 40 km/h and 60 km/h respectively.

The relative motion between cars and TX, results in random frequency modulation due to different Doppler Shifts on each of the multipath components. If the Doppler Shift is given by [34]:

(5) $$\mathrm{fd}=\;\frac{1}{2\pi }\cdot \frac{\Delta \emptyset }{\Delta t}=\frac{v}{\lambda }\cdot \mathrm{Cos}\theta$$

the theoretical fd_max_ (40 km/h) = 218.52 Hz and f_dmax_ (60 km/h) = 327.78 Hz, when Cosθ = 1.

When PA is traveling at a constant velocity of 40 km/h, the simulated fd_max_ were reported between −207,821 and 213.33 Hz, while for 60 km/h the simulated fd_max_ were reported between −311.72 and 319.99 Hz. The reported fd positive values is an indicator that PA is moving directly toward the TX and the apparent received frequency is defined by: f = (fc + fd) Hz, meanwhile negative fd values means that PA is moving directly away the TX and the apparent received frequency is f = (fc − fd) Hz. According to this definition and simulation results, the received signal spectrum will have components in the range f = (5.9 × 10^9^ − 311.72 to 5.9 × 10^9^ + 319.99) Hz. Additional simulation shows that the signal spectrum components for PB and PC (traveling at constant velocity of 60 km/h) are in the range of f_PB_ = (5.9 × 10^9^ − 297.05 to 5.9 × 10^9^ + 299.42) Hz and f_PC_ = (5.9 × 10^9^ − 148.80 to 5.9 × 10^9^ + 192.65) Hz respectively.

**Figure 13:**
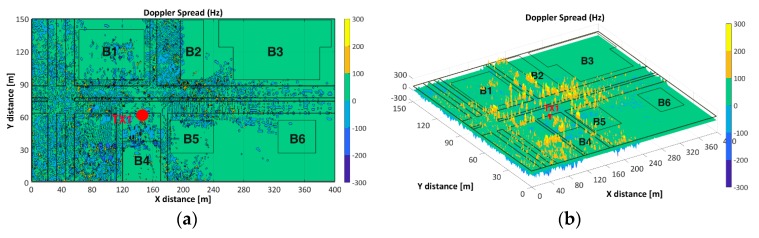
Doppler Spread (B_D_) for TX1: (**a**) velocity of 40 km/h, (**b**) velocity of 60 km/h.

Figure 13a,b show the TX1-B_D_ for velocities of 40 km/h and 60 km/h respectively. Higher B_D_ values are observed in the vicinity of the TX1 and higher B_D_ values are depicted for velocity of 60 km/h. Figure 13a clearly illustrates the positive and negative range of received frequencies when the PA is traveling at 40 km/h. Factors such as the relative velocity and direction of motion respect to TX, have an impact on the apparent received frequency which impact B_D_, T_C_ and fd values, as aforementioned.

## 4. Statistical Analysis

The fading channel in vehicular environments could be a combination of different fading channel types that changes over distance, time, and mobility. Different fading models have been proposed and utilized in literature as Gamma, Lognormal, Rician [23], Rayleigh, Nakagami, Weibull, composite fading models as Weibull-Gamma [36], Nakagami-Lognormal, K distribution (mixture of Rayleigh and Gamma), mixtures of Gamma distributions [37], etc. In this multipath environment, identification of some statistical fading model able to fit and characterize the fading level of this urban scenario is required, as well as the adoption of a descriptive statistic to measure it.

To validate the closeness of the theoretical distribution (Gamma, Lognormal, Weibull, etc.) function to the empirical distribution function (RSS 3D-RL) we used the Anderson-Darling (AD) test, which was another common test along with Kolmogorov-Smirnov(K-S), Cramer-von Mises (C-vM), Chi_Square(C-S), etc. We evaluate the Goodness-of-fit (GOF) for nonparametric data sets. The AD statistic provides robustness and flexibility compared to other GOF measures because it gives more weight to the tail of the distribution, and it has been employed to quantify the difference between the observed and expected values [38], as a type of divergence or discrepancy measurement.

In applications such as Cognitive Radio, a proposed AD sensing has much higher sensitivity to detect a transmission signal than energy detector-based sensing, especially for small samples [39]; however, in applications such as hydrological statistics, the results show most powerful GOF results for K-S and C-S test than AD [40]. On the other hand, one of the descriptive statistics to measure data variability is the squared coefficient of variation (SCV) defined as [41]:

(6) $$\mathrm{SCV}=\frac{\mathrm{Var}}{{µ}^{2}}=\frac{E\left({\left(x-µ\right)}^{2}\right)}{{µ}^{2}}=\frac{E\left(x^{2}\right)}{{\left(E\left(x\right)\right)}^{2}}-1$$

where x is a random variable and E (·) is expectation operator. According [42], for a given distribution of strength, *a*, of a received signal, the amount of fading (AF) is defined by the ratio of the variance of the received energy to the square of the mean of the received energy as follows:

(7) $$\mathrm{AF}=\frac{\mathrm{Var}\left(a^{2}\right)}{{\left(E\left(a^{2}\right)\right)}^{2}}$$

According to Equations (6) and (7), the SCV of received signal power characterizes the amount of fading (AF) of signal power and will be used in this section to describe the data variability and AF.

**Figure 14:**
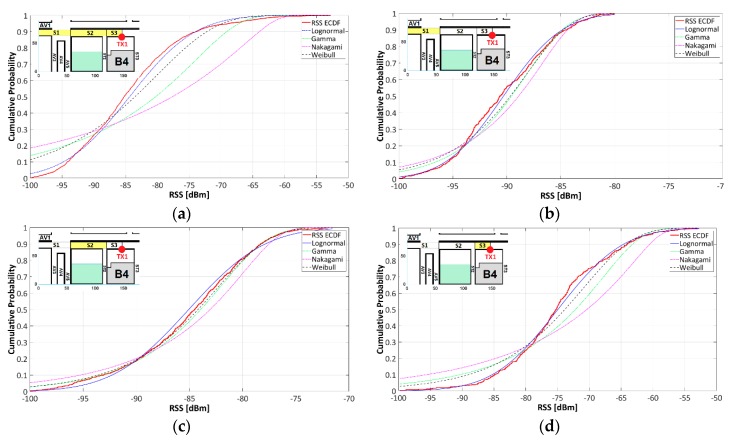
CDF for TX1 (S1-to-S3) along AV2. (**a**) S1, S2, S3 (x: 1 m to 146 m); (**b**) S1 (x: 1 m to 60 m); (**c**) S2 (x: 60 m to 120 m); (**d**) S3 (x: 120 m to 164 m).

Figure 14 depicts the TX1-RSS Cumulative Distribution Function (CDF) along the AV2 (refer to Figure 9b) and its statistical comparison with theoretical distributions widely used in the description of multipath fading environments. Complementary to this, Table 4 summarizes: (a) the 3D-RL data Squared Coefficient of Variation (SCV) a statistical measure of the data dispersion level; (b) the GOF between the theoretical distributions and the 3D-RL data using the AD test with 5.0% for a significance level of the hypothesis (H_0_) test; and (c) the input parameter (shape) for the tested distributions. The TX1-RSS coverage area along AV2 was divided into 6 segments: S1 to S3 (left to TX1) and S4 to S6 (right to TX1) where S1 is symmetric and equidistant with S6, S2 with S5 and S3 with S4. This kind of segmentation was chosen for three reasons: (a) to consistently identify the statistical differences between symmetric and equidistant areas (spatial considerations) to TX1; (b) because of reasonable balance between a maximum segment-length and a significant statistical fit when compared with theoretical distributions; and (c) to organize the data into homogeneous segments, avoiding as much as possible extreme 3D-RL RSS values.

Figure 14a and Figure 9b,c show the CDF comparison between the ECDF of S1(60 m), S2(60 m), and S3 (26 m) with Gamma, Lognormal, Nakagami and Weibull distributions. Table 4, items (a) and (c) show that S1 and S3 can be described in terms of Lognormal distribution while S2 is better fitted by Weibull distribution. The SCV of each individual segment is significant lower than the joint segment which is an indicator of data homogeneity that can lead to consistent statistical GOF test. Analogous statistical behavior was obtained for the symmetric and equidistant segments S6, S7 and S8 (Table 4, items f, g, h).

Figure 14d depicts the CDF for the joint data segment S1 to S3 (146 m), and its comparison with Gamma, Lognormal, Nakagami, and Weibull distributions where the best fit is observed for the Lognormal distribution; however, the AD-GOF test resulted in the rejection of the null hypothesis (H_0_) which means that the RSS statistical behavior of this segment does not fit with any of the tested distribution. The GOF test results for Gamma, Lognormal, Nakagami and Weibull distributions are registered in Table 4, item d. This joint segment S1 to S3 is characterized by a high data dispersion level measured by the SCV index (34.268), indicator of heterogeneity in the RSS data. This heterogeneity could be a possible factor that causes the rejection of the null hypothesis. Consequently, it makes necessary an analysis with smaller segments where the RSS data is distributed homogenously. Analogous results were obtained for the joint segment S5 to S6 (144 m) according the results in Table 4, item e.

When the GOF test indicates the non-rejection of the null hypothesis for two or more distributions, the best distribution fit will be defined for the lower “AD-statistic” given that this statistic represents a divergence or discrepancy measurement [43]. The input parameter (shape)—estimated from the 3D-RL RSS data using Maximum Likelihood Estimation MLE—that characterizes the Gamma, Lognormal, Nakagami and Weibull distributions, shows slight variations for the shape parameter when the individual segments are compared; for the joint segments these parameters were not reported given the lack of fit between the data and the proposed distributions. 

## 5. Measurements Results

To have a complete characterization of the considered scenario, the radio wave electromagnetic propagation analysis presented in the above sections has been complemented with a network parameter assessment with the aid of real measurements implemented in the same scenario. The description of the campaign of measurements was presented in Section 2.3.

### 5.1. Throughput Analysis in V2V and V2I Links

**Figure 15:**
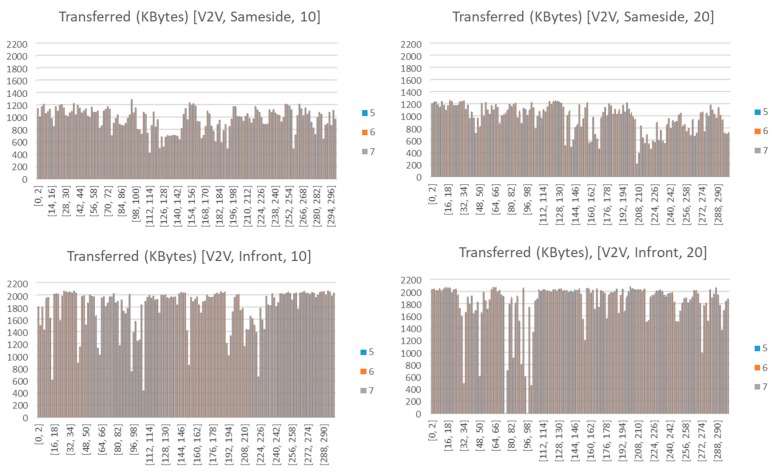
Disaggregation of the Kbytes transferred by each thread when considering a communication between vehicles (V2V) located on the same side (**top**) or on the opposite side (**bottom**) and different bandwidths (10 Mbps -left-, and 20 Mbps -right-).

During the experiments, the analysis of the traffic exchange shows that best results correspond to the communication V2V, when comparing V2V and V2I. This is due to the orientation of the antennas, which were placed, with the aid of a magnet, perpendicular to the streetlight, as depicted previously in Figure 5e–g. This causes the loss of messages since it was not possible to get good coupling between the antennas located on the roof of the vehicles and those located in the streetlights. This fact does not prevent communication, but the quality of the link decreases markedly. In the same way, the differences of the link communication when vehicles and infrastructure are located on the same side of the avenue, and when they are not, have been analyzed. The orientation of the vehicles affects several transmission parameters: relative speed, distance and obstacles. During experimentation, the distance between each vehicle was higher when they were located on the same side of the avenue than when they were in opposite lanes (see Figure 2), and the presence of garbage containers between the cars also disturbed the communication. Three communication threads were used (3, 5 and 7) to analyze if the use of simultaneous transmission of several threads affects the success of the communication. However, as can be observed from Figure 15, it has no visible relevance. Thus, the rest of the figures will just show the average values. Figure 15 depicts the effective throughput transferred by each thread, according to the type of communication (V2V or V2I) and the bandwidth (10 or 20 Mbps) of each thread. As can be observed, the number of Kbytes transferred is quite similar. Each communication has a duration of 300 s.

It is observed from Figure 15 that the throughput varies over time. That is due to the passage of vehicles along the avenue. The larger the vehicles (trucks and buses), the longer the duration of the fading and the greater of its intensity.

**Figure 16:**
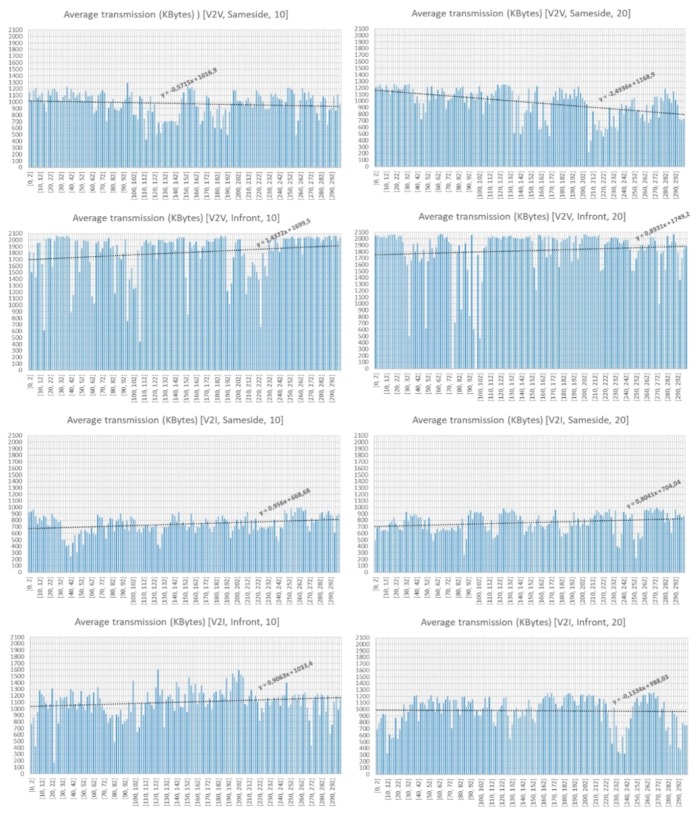
Average transmission. Number of Kbytes received.

**Figure 17:**
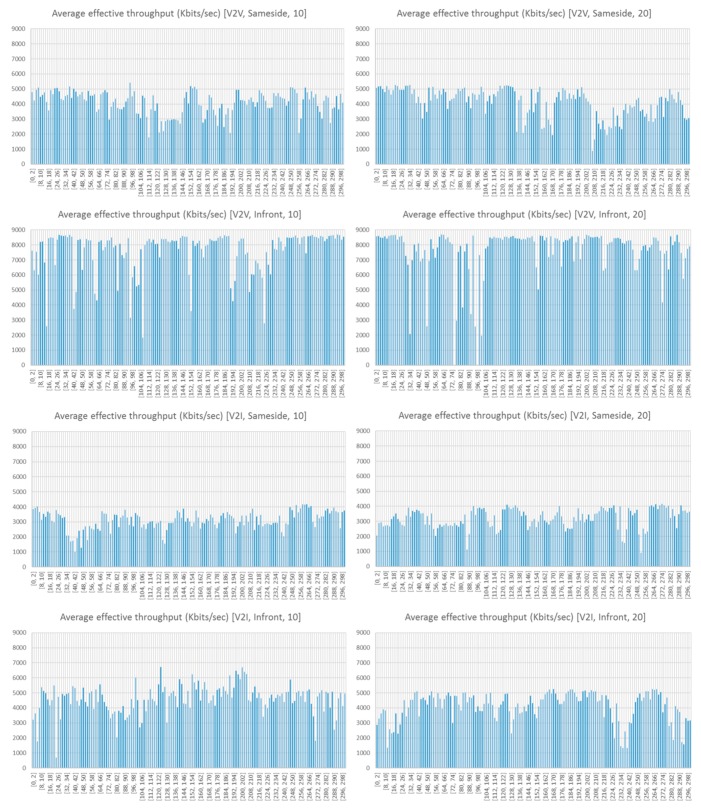
Average effective throughput received.

Figure 16 shows the average values of the Kbytes transferred, while Figure 17 shows the average effective throughput received. As it can be observed from Figure 16, the location on the same side of the antennas (either V2V or V2I) results in a lower data transmission rate. The best transmission results are obtained when the transceivers (V2V or V2I) are located on the opposite sides (in front). V2V communication provides an average data transmission noticeably better than V2I. However, the use of a greater bandwidth (10 and 20 Mbps) on the transmission threads does not seem to influence too much the number of bytes received. 1808 Kbytes are received on the V2V-Infront case, while 1102 Kbytes are received on the V2I-Infront case. Worst values are obtained on V2I-Sameside (741 Kbytes) and V2V-Sameside (974 Kbytes). The analysis of the effective throughput received (see Figure 17) shows a similar pattern, so we can conclude that V2V-Infront is the best configuration and V2I-Sameside provides the worst results. 

At this point, we cannot consider whether it is better or not to choose a lower or higher bandwidth on transmission, but the analysis of PER reveals that it is better to opt for a lower transmission rate, since it guarantees a lower rate of losses, which results in a better functioning of the system.

### 5.2. Packets Loss and Jitter

**Figure 18:**
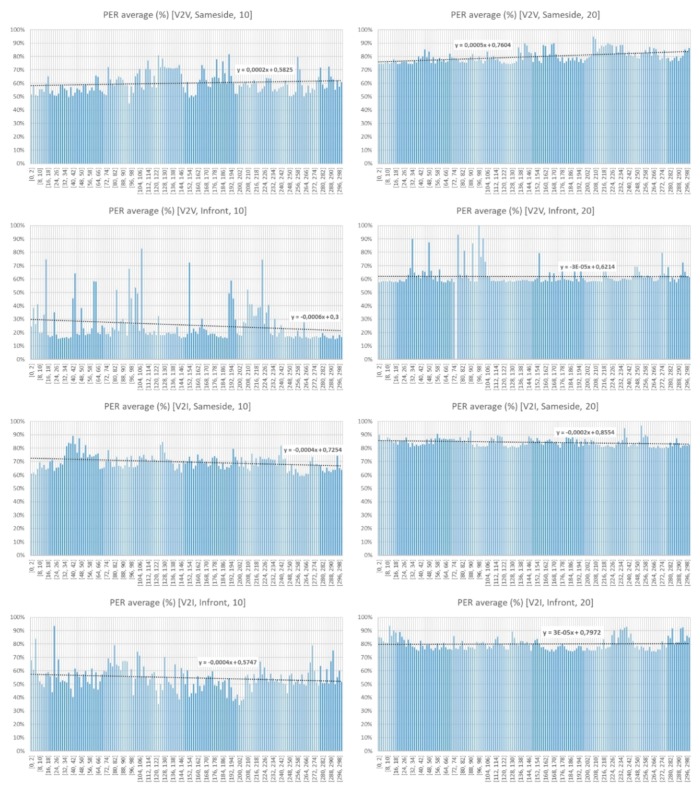
Average packet error rate (PER) observed.

Figure 18 shows that the PER rate is clearly worse when we choose a higher bandwidth (20 Mbps) in each transmitter thread. PER average, when considering the V2I scenario, is higher, as the bandwidth required increases. This is due to the higher number of collisions observed. The PER average increases with distance. The graphs corresponding to the same side show lower PER rates than those of Infront, where vehicles are closer.

**Figure 19:**
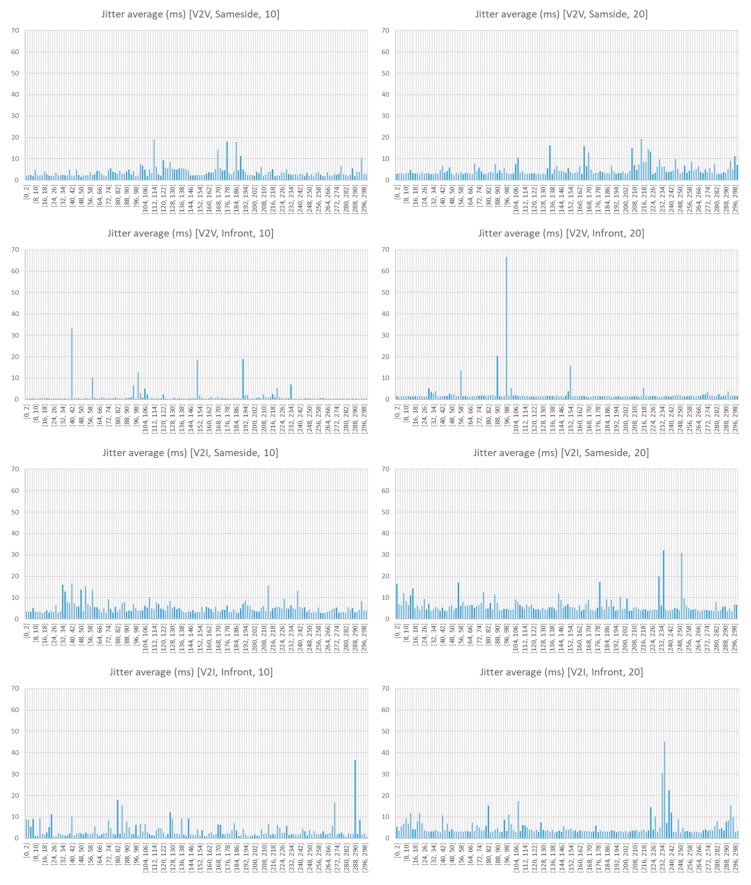
Average jitter (ms) observed.

Figure 19 shows the jitter (deviation on the time) observed. Jitter values are higher when transceivers are located on the same side and increase as the transmission bandwidth does. If the communication channel becomes saturated, and that is something that can happen at 10 Mbps bandwidth, the increase of the transmission bandwidth should not improve the speed, but it could harm the jitter. Therefore, lowering the transfer rate itself would mean an improvement in the effective throughput.

Thus, we can conclude from the observation of the measured data that communication between vehicles (V2V) located in opposite sides (called Infront in the figures) transmitting with rates not too high (10 Mbps) is preferable. V2V-Infront-10 is the operation configuration that offers better results, and therefore, the one that should be considered preferable.

## 6. Application

To show a possible usage of the characterization of V2V and V2I communication links in these types of scenarios, a simple application has been developed with the sole purpose of notifying the driver of traffic incidents in the simplest possible way, trying not to trouble him/her. For such a purpose we have built an Android-based mobile application following an MVC (Model-View-Controller) software pattern. The application, making use of the BATMAN-ADV protocol previously described, receives messages concerning traffic issues detected by other vehicles and by infrastructure. The application is just a simple traffic messaging tool that allows the exchange of messages in real time among the vehicles that take part of the vehicular network (VANET) at a given instant. In the same way, the application can warn other drivers of any incident detected in or near the vehicle by sending the corresponding message/s.

**Figure 20:**
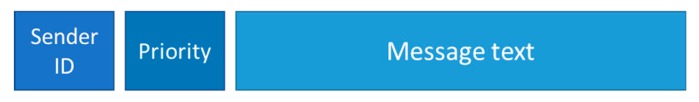
Packet structure.

Given that this article focuses exclusively on the communication of V2V and V2I, we have simulated the traffic issues/messages to validate the correct behavior of the system. We inject in the system synthetic messages announcing the arrival of emergency vehicles, the change of traffic lights, the existence of dense traffic, the presence of a damage vehicle, and even more. Some of those messages are sent by the infrastructure (e.g., change of traffic lights) and others by the vehicles (e.g., emergency vehicles) but in both cases, a message is exchanged with the other nodes of the network. We have defined a simple packet structure (see Figure 20) that includes the sender identification (car number plate), the level of priority to establish the ordering on the presentation of the messages received and their persistence on the visualization screen, and finally, the message to be displayed. The application is responsible for not displaying repeated messages that have a previously received message identifier. The application uses the sender’s ID field, which contains a unique identifier of the sender and a counter of the messages send by this sender.

**Figure 21:**
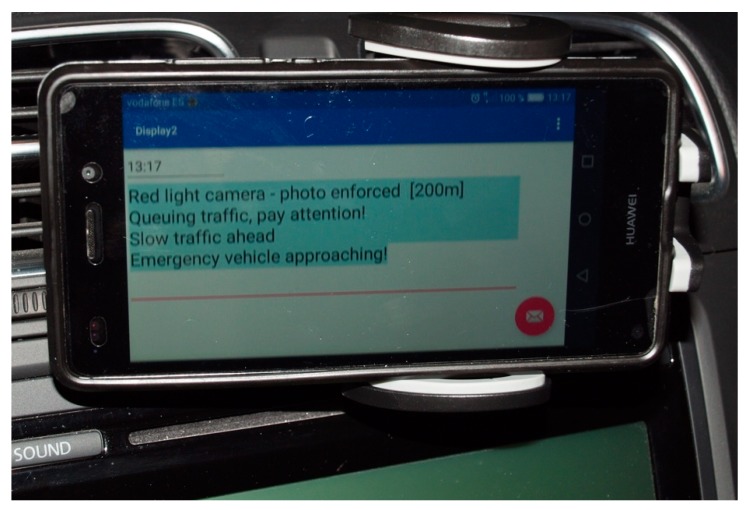
GUI of the application.

Figure 21 shows the graphic user interface of the application. The App allows the selection of the text size, and allows the messages to be audible with the help of a voice synthesizer (TextToSpeech) wthin the device.

## 7. Conclusions and Future Work

A complete characterization in terms of physical and network parameters of an urban vehicular scenario has been presented in this work, showing the great influence of the morphology and topology of the scenario in V2V and V2I communication links.

Radio propagation analysis, with the aid of a hybrid deterministic code based on 3D RL, enables an accurate estimation of urban large-scale and small-scale parameters such as RSS, RMS delay spread, CB, PDP and Doppler Shift at 5.9 Ghz frequency. A large urban scenario (400 m × 150 m × 22 m) has been characterized with high level of detail which permits accurate results of radio propagation parameters that could be replicated in similar environments and be useful for radio-planning WSNs designers in V2I communication systems.

Results show the impact of factors as distance, spatial location, LoS conditions, frequency and vehicle speed, in the V2I small and large-scale parameters. The large-scale analysis shows a variable path loss exponent and RSSI dispersion, while a coverage map displays consistent RSS values above the RST for TX1 and TX2 for an approximate distance of 90 m under LoS conditions. The presence of trees causes QLoS and intense dispersive signal conditions which is evident in the PDP mainly at closest points to the TX, while NLoS conditions are caused by the obstruction of the radiated signal by buildings. The highest RMS delay spread was registered near the TX and along the line of trees on the main avenues, where lower CB values are an indicator of channel availability for other transmitters. The relative motion of the cars with respect to the TX, measured by the Doppler Shift on each of the multipath components, shows its highest values for vehicles traveling near the TX. Considering the aforementioned factors, a four-TX configuration is suggested to provide V2I wireless communication with consistent RSS values above the RST and almost complete scenario coverage.

Statistical analysis gives insight into the importance of the scenario segmentation, where homogeneous clusters of information can lead to identification of the variations related to distance and spatial location, to get consistent and significant results for analysis and further simulations. Lognormal, Gamma and Weibull distributions can describe the fading RSS behavior when a GOF test is performed. Parameters such as PLE, SCV, AF, and distribution shape factor cannot be assumed constant given the non-stationary nature of the vehicular communications channel.

Network layer parameters analysis in the urban vehicular scenario shows that the orientation of the antennas has a significant influence on the V2V and V2I communication links, being able to decrease considerably the quality of the link in some cases for V2I. Vehicles in movement in the road, as well as larger vehicles, such as trucks and buses, affect the link significantly, causing throughput variability over time. Parameters such as bandwidth, PER and jitter are also important and must be considered. Experiments show that PER and jitter parameters are worse when a higher bandwidth is chosen. It can be concluded that, in some cases, lowering the transfer rate itself would mean an improvement in the effective throughput. 

## Figures and Tables

**Table 1:** Scenario reference.

Reference	Abbreviation	Coordinates (x, y, z) m
Main Avenues	AV1/AV2	(x, 83, 0)/(x, 69, 0)
Streets	ST1/ST2/ST3	(161, y, 0)/ (117, y, 0)/ (175, y, 0)
Transmitter antenna (TX)	TX1/TX2	(146, 63, 3.5)/(281, 63, 3.5)
Receiver antenna (RX)	RX	(x, y, 1.5)
Buildings	B1, B2, B3, B4, B5, B6	Not applicable.

**Table 2:** Simulation parameters.

Parameters	Values
**TX1, TX2**:	
$P_{t}\;$*/$\mathrm{Gain}(G_{t}$)/Frequency/Height/ polarization.	0 dBm/0 dB/5.9 Ghz/3.5 m omnidirectional.
**RX**: RST **/$\mathrm{Gain}\left(G_{r}\right)$/Frequency/Height.	−100 dBm/0 dB/5.9 Ghz/1.5 m
/polarization.	omnidirectional.
**3D-RL**: horizontal and vertical angular resolution	π/180 rad
Angular resolution of diffracted rays.	π/20 rad
Maximum permitted reflections.	7 hops
Cuboid segmentation for analysis.	1 m^3^ (1 × 1 × 1) m
**Scenario**: dimension.	(400 × 150 × 22) m

Pt *: Power transmitted, RST ** (Received Signal Threshold).

**Table 3:** Path loss exponent (n) and standard deviation (STD) for TX1.

Description	PLE (n)	STD (σ) [dB]
**(a) Along AV1 (y = 81 m)**		
x: (0 to AV5/AV5 to TX1/TX1 to TX2/TX2 to 400) m	(2.8/2.5/2.8/4.3)	(30.8/13.1/25.1/44.6)
**(b) Along AV2 (y = 70 m)**		
x: (0 to AV5/ AV5 to TX1/TX1 to TX2/ TX2 to 400) m	(2.6/2.4/2.5/5.0)	(20.1/11.3/18.0/44.0)
**(c) Along ST1 (x = 161 m)**		
y: (AV2 to 150) m	2.40	9.21
**(d) Along ST2 (x = 116 m)**		
y: (LoS: 1 to 21/NLoS: 21 to AV1) m	(4.13/ 2.48)	(11.32/8.11)
**(e) Along ST3 (x = 178 m)**		
y: (LoS: 1 to 21/NLoS: 21 to AV1) m	(4.21/2.28)	(15.56/3.36)

**Table 4:** Data dispersion measurements and GOF test for TX1.

Description	SCV **	n ***	Lognormal	Gamma	Nakagami	Weibull
**(a)** S1: (x: 1 to 60) m	1.126	3.099				
CDF-GOF: AD * (Hypothesis test/statistic) Input parameter: Shape			**F**/1.817 0.072	T/8.029 1.210	T/19.552 0.391	T/7.485 1.058
**(b)** S2: (x: 60 to 120) m	1.715	2.345				
AD (Hypothesis test/statistic) Shape factor			T/3.951 1.136	T/3.558 0.842	T/23.013 0.289	**F**/1.936 0.870
**(c)** S3: (x: 120 to 146) m	8.473	2.449				
AD (Hypothesis test/statistic) Shape factor			**F**/1.051 3.466	T/20.25 0.396	T/44.186 0.139	T/7.9071 0.541
**(d)** S1, S2, S3, S4 (x: 1 to 146) m AD (Hypothesis test/statistic)	34.268	2.764	T/11.261	T/Inf	T/Inf	T/70.462
**(e)** S5, S6, S7 (x: 146 to 290) m AD (Hypothesis test/statistic)	27.526	3.041	T/0.313	T/4.544	T/46.353	T/2.495
**(f)** S6: (x:146 to 170) m	6.691	2.377				
AD (Hypothesis test/statistic) Shape factor			**F**/0.878 3.987	T/19.259 0.479	T/46.353 0.161	T/8.437 0.609
**(g)** S7: (x: 170 to 230) m	1.826	2.284				
AD (Hypothesis test/statistic) Shape factor			**F**/1.639 1.507	T/7.946 0.827	T/34.403 0.282	T/4.809 0.855
**(h)** S8 (x: 230 to 290) m	1.225	3.610				
AD (Hypothesis test/statistic) Shape factor			F/1.342 0.013	T/4.658 1.149	T/18.919 0.370	T/4.668 1.029

AD *: Anderson-Darling test, SCV ** (Squared Coefficient of Variation), n *** (Path loss exponent). T = True, F = False (for hypothesis test)
